# Japanese nationwide survey of hypophosphatasia reveals prominent differences in genetic and dental findings between odonto and non-odonto types

**DOI:** 10.1371/journal.pone.0222931

**Published:** 2019-10-10

**Authors:** Rena Okawa, Kazuma Kokomoto, Taichi Kitaoka, Takuo Kubota, Atsushi Watanabe, Takeshi Taketani, Toshimi Michigami, Keiichi Ozono, Kazuhiko Nakano

**Affiliations:** 1 Department of Pediatric Dentistry, Osaka University Graduate School of Dentistry, Osaka, Japan; 2 Department of Pediatrics, Osaka University Graduate School of Medicine, Osaka, Japan; 3 Division of Clinical Genetics, Kanazawa University Hospital, Ishikawa, Japan; 4 Department of Pediatrics, Shimane University Faculty of Medicine, Shimane, Japan; 5 Department of Pediatric Nephrology and Metabolism, and Department of Bone and Mineral Research, Osaka Medical Center and Research Institute for Maternal and Child Health, Osaka, Japan; School of Dentistry, University of Sao Paulo, BRAZIL

## Abstract

Hypophosphatasia (HPP) is a rare and intractable metabolic bone disease caused by mutations in the *ALPL* gene. Here, we undertook a nationwide survey of HPP in Japan, specifically regarding the prominent genetic and dental manifestations of odonto (*n* = 16 cases) and other (termed “non-odonto”) (*n* = 36 cases) types. Mean serum alkaline phosphatase (ALP) values in odonto-type patients were significantly greater than those of non-odonto-type patients (*P*<0.05). Autosomal dominant and autosomal recessive inheritance patterns were detected, respectively, in 89% of odonto-type and 96% of non-odonto-type patients. The *ALPL* “c.1559delT” mutation, associated with extremely low ALP activity, was found in approximately 70% of cases. Regarding dental manifestations, all patients classified as odonto-type showed early exfoliation of the primary teeth significantly more frequently than patients classified as non-odonto-type (100% vs. 56%; *P*<0.05). Tooth hypomineralisation was detected in 42% of non-odonto-type patients, but not in any odonto-type patients (0%; *P*<0.05). Collectively, these results suggest that genetic and dental manifestations of patients with odonto-type and non-odonto-type HPP are significantly different, and these differences should be considered during clinical treatment of patients with HPP.

## Introduction

Hypophosphatasia (HPP) is a rare, metabolic bone disease caused by mutation of the *ALPL* gene, which encodes tissue-nonspecific alkaline phosphatase (ALP) in bone osteoblasts, and liver, kidney, and skin fibroblasts. *ALPL* mutations lead to a reduction in ALP activity [1–6], characterised by bone hypomineralisation (decreased mineral content) and/or early exfoliation of the primary teeth [1,4,7]. HPP is diagnosed by one or both of these main symptoms, as well as low serum ALP values [8]. Early tooth exfoliation often occurs in the anterior mandibular regions before the age of 4 years, caused by disruptions in the formation of root cementum and poor attachment of the periodontal ligament to the alveolar bone [7,9,10], such that exfoliated teeth show no signs of root resorption.

HPP is classified into six subtypes depending on the time of diagnosis and symptoms: perinatal severe (in utero and at birth), perinatal benign, infantile (before 6 months of age), childhood (after 6 months of age to 18 years old), adult (after 18 years old) and odonto (at all ages after tooth eruption, with only dental symptoms) [1,3–6]. Symptoms are typically more severe with earlier-aged diagnoses, except for perinatal-benign and odonto types [1,3–6]. Patients with severe-type HPP, such as perinatal or infantile types, receive general care from medical doctors before or soon after birth. However, in less-severe types, such as odonto or childhood types, dental signs are often the first clues toward a diagnosis [7,11,12].

Severe-type HPP is considered to be inherited in an autosomal recessive (AR) manner, whereas milder types are AR or autosomal dominant (AD) [1,3,7,13], and some AD *ALPL* mutations have been reported to have a dominant-negative effect [14]. The frequency of severe-type HPP is estimated to be 1/100,000 [1,2,4]. A common *ALPL* mutation among Japanese patients is “c.1559delT”, which results in extremely low enzyme activity [13,15–17]. Homozygous “c.1559delT” mutations are responsible for the severe-type HPP [13,15–17], whereas heterozygous mutations are estimated at 1/480 [17].

HPP is a progressive disease, and patients with odonto-type HPP without bone symptoms can progress to childhood-type or adult-type HPP with age upon presentation of bone symptoms [11,18]. Therefore, screening for patients with only dental manifestations is important for an early diagnosis. Enzyme replacement therapy (ERT) has extended life expectancies among HPP patients, particularly those with severe-type HPP, such as perinatal-severe type, which is fatal within the first year of life due to respiratory failure [1–6]. Initiated in Japan in 2015, ERT enables many patients who would not normally survive until tooth eruption to receive dental management [19–27]. However, the dental symptoms of patients with severe-type HPP who receive ERT are different from those of patients with mild-type HPP without ERT; albeit, there are only a few reports regarding dental changes associated with ERT.

Under normal conditions, the first primary teeth emerge in the oral cavity at around 8 months of age, and primary dentition is usually complete by 2.5 to 3 years [28]. Tooth exfoliation then usually commences at the age of 6 years when the permanent teeth start to erupt, with permanent dentition established around 12 to 14 years [28]. However, knowledge of these events in patients with HPP is limited, and previous studies have focussed only on the early exfoliation of primary dentition, with fewer considerations of the effects of other dental manifestations, such as severe periodontal conditions or hypomineralisation of the enamel and dentine.

In the present study, we conducted a nationwide survey in Japan regarding genetic and dental manifestations of HPP, focusing on the differences between odonto and non-odonto types.

## Materials and methods

### Collection of clinical records of subjects with HPP

In the nationwide survey, we collected clinical records to analyse the oral manifestations of patients diagnosed with HPP. A total of 619 dental clinics among general hospitals with dentistry departments, including 28 university dental hospitals with departments of paediatric dentistry, were invited to participate. Clinics were sent questionnaires regarding the number of HPP cases encountered over a recent 5-year period, from 2012 to 2017 (S1 Fig). When clinics indicated that they had treated HPP cases, we proceeded with a second questionnaire regarding the clinical dental findings of these cases (S2 Fig). We also collected clinical records of 14 HPP cases who came to our clinic.

### Clinical analyses

Patient information from the second questionnaire (S2 Fig) was assessed and recorded in terms of chronological age at the time of the first and last examination, gender, phenotype, medical information at diagnosis (serum ALP value [Japanese Society of Clinical Chemistry method [29]], urinary phosphoethanolamine, *ALPL* mutation), family medical and dental histories, treatment with ERT, reason for visiting dental clinic, collaboration with medical and dental practitioners, presence of early exfoliation of primary and permanent teeth (positions and age at loss), other dental abnormalities (periodontal conditions, hard tissue conditions, eruptions, and occlusion), dental treatments, and other systemic anomalies.

### Ethical considerations

This study was conducted in full adherence to the Declaration of Helsinki (64th World Medical Association General Assembly, Fortaleza, Brazil, 2013), and study protocols were approved by the Ethics Committee of Osaka University Graduate School of Dentistry (approval no. H29-E15). All data were fully anonymised before they were accessed in this study, and the ethics committee waived the requirement to obtain informed consent from patients. Because this was a retrospective observational study using only existing medical records, informed consent was obtained via opt-out on our hospital website. Patients who opted out were excluded from the study.

### Statistical analysis

Statistical analyses were conducted using GraphPad Prism 7 (GraphPad Software Inc., La Jolla, CA, USA). Intergroup differences of serum ALP values were analysed using analysis of variance (ANOVA) with Bonferroni correction. Results were considered significantly different at *P*<0.05. Differences in inheritance patterns were assessed by the Chi-square test. Fisher’s exact test was used to analyse differences between groups in terms of dental conditions and the early exfoliation of primary teeth.

## Results

### Number of HPP cases

A total of 619 dental clinics in general hospitals with dentistry departments, including 28 university dental hospitals with paediatric dentistry departments, were invited to participate. Of these, 381 clinics (61.6%) completed the first questionnaire regarding the number of HPP cases encountered over a recent 5-year period (2012 to 2017; S1 Fig). Fifty-four HPP cases were reported by 26 clinics (4.2%). These clinics then received a second questionnaire regarding clinical dental findings (S2 Fig). Finally, we obtained the clinical records of 38 HPP cases (22 clinics [3.6%]; 12 dental clinics in general hospitals with dentistry departments and 10 university dental hospitals with paediatric dentistry departments). We included an additional 14 HPP cases from our clinic, and analysed a total of 52 HPP cases (26 males, 26 females).

Table 1 summarises the medical information of all cases collected in the present study. The most frequent phenotype reported was odonto type, followed by infantile, perinatal severe, childhood, and perinatal benign. Two cases in the odonto-type group shifted to the childhood-type group because of the emergence of bone symptoms with aging.

**Table 1:** General information regarding patients with HPP.

Phenotype	Total no. of patients (Rate for all patients)	Ages of HPP diagnosis Mean ± SEM (months) [median]	Total no. of patients who received ERT (Rate for all patients in each phenotype)	Age at dental examination Mean ± SEM (years) [median]	
				First	Last
Perinatal severe	9 (17%)	1.1 ± 1.1 [0]	9 (100%)	1.8 ± 0.3 [1.5]	4.0 ± 0.8 [3.8]
Perinatal benign	6 (12%)	0.6 ± 0.6 [0]	3 (50%)	3.7 ± 1.0 [4.4]	8.6 ± 2.5 [7.4]
Infantile	13 (25%)	9.8 ± 6.8 [2.0]	10 (77%)	2.6 ± 1.1 [1.3]	9.5 ± 0.9 [9.7]
Childhood	8 (15%)	59.1 ± 15.9 [49.0]	3 (38%)	6.7 ± 1.4 [7.2]	9.5 ± 2.5 [7.0]
Odonto	16 (31%)	37.0 ± 4.0 [40.5]	1 (6%)	3.5 ± 0.6 [2.6]	6.0 ± 0.7 [5.2]
Total	52 (100%)	23.9 ± 4.4 [11.0]	26 (50%)	3.4 ± 0.4 [2.0]	7.3 ± 0.7 [6.1]

ERT, enzyme replacement therapy; HPP, hypophosphatasia; SEM, standard error of the mean

### Patient background

**Fig 1:**
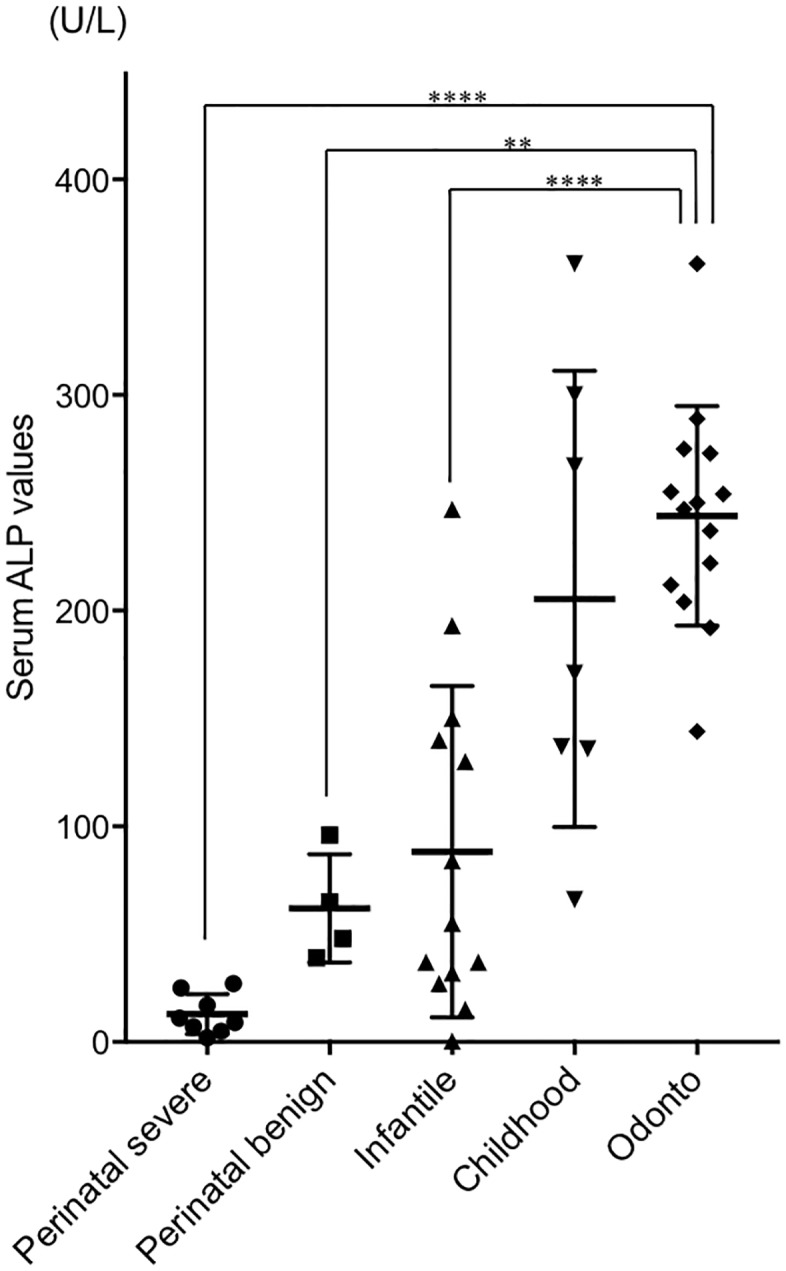
Distribution of serum ALP values of each clinical type. Significant differences were determined using ANOVA with Bonferroni correction. **P<0.05 and ****P<0.0001 versus odonto type.

The average age at diagnosis in patients with odonto-type HPP was 37.0 months (range, 13.0 to 71.0 months), compared with 59.1 months for patients with childhood-type HPP (*P* = 0.0587; Table 1). Fifteen cases of odonto-type (93.8%) and two childhood-type (25%) HPP were diagnosed by medical doctors after referral by dentists who suspected HPP based on typical dental findings of early primary tooth exfoliation. Fig 1 shows the distribution of serum ALP values for patients in each clinical type. The mean serum ALP value for patients with odonto-type HPP was 243.9 U/L, which was significantly greater than those for patients with perinatal-severe (12.9 U/L), perinatal-benign (62.0 U/L), and infantile (88.3 U/L) types (*P*<0.05). Of the 26 cases (50%) who received ERT, there were nine patients (100%) with perinatal-severe-type and one with odonto-type HPP (Table 1).

### Genetic features

*ALPL* mutation analyses were carried out in 39 cases, and information regarding the mutations was obtained for 35 cases (Table 2). The “c.1559delT, p.Leu520Argfs*86 in exon 12” mutation was detected in 24 cases (69%), followed by “c.979T>C, p.Phe327Leu in exon 9” in five cases (14%), and “c.550C>T, pArg184Trp in exon 6” in four cases (11%); of these last four, three were single mutations for patients with odonto-type HPP. Among patients with non-odonto-type HPP, a homozygous “c.1559delT” mutation was detected in six cases (26%), compound heterozygous mutations were detected in 17 cases (71%), and a heterozygous mutation was detected in one case (4%). One odonto-type case had a compound heterozygous mutation for “c.1559delT”. One patient with childhood-type HPP, who had “c.572A>G, p.Glu191Gly of exon 6” and “c.1559delT, p.Leu520Argfs*86 of exon12” mutations, was originally diagnosed with odonto-type HPP.

**Table 2:** *ALPL* mutations in each clinical type.

Phenotype	Genotype						Number of patients
	exon	allele	Residual Activity % WT	exon	allele	Residual Activity % WT	
Perinatal severe	6	c.550C>T, p.Arg184Trp	0.6	**12**	c.1559delT, p.Leu520Argfs*86	0	1
	6	c.563C>T, p.Ser188Pro	ND	**12**	c.1559delT, p.Leu520Argfs*86	0	1
	12	c.1436A>G, p.Tyr479Cys	ND	**12**	c.1559delT, p.Leu520Argfs*86	0	1
	12	c.1471G>A, p.Gly491Arg	NR	12	c.1471G>A, p.Gly491Arg	NR	1
	**12**	c.1559delT, p.Leu520Argfs*86	0	**12**	c.1559delT, p.Leu520Argfs*86	0	4
Perinatal benign	7	c.678G>T, p.Met226Ile	ND	**12**	c.1559delT, p.Leu520Argfs*86	0	1
	9	c.979T>C, p.Phe327Leu	NR	**12**	c.1559delT, p.Leu520Argfs*86	0	2
Infantile	5	c.319G>A, p.Val107lIe	ND	12	c.1403C>T, p.Ala468Val	NR	1
	5	c.407G>A, p.Arg136His	33.4	**12**	c.1559delT, p.Leu520Argfs*86	0	1
	6	c.572A>G, p.Glu191Gly	NR	**12**	c.1559delT, p.Leu520Argfs*86	0	1
	7	c.670A>G, p.Lys224Glu	43	11	c.1276G>T, p.Gly426Cys	18.5	1
	9	c.979_980delCTT, F310del	ND	**12**	c.1559delT, p.Leu520Argfs*86	0	1
	9	c.979T>C, p.Phe327Leu	72	**12**	c.1559delT, p.Leu520Argfs*86	0	1
	10	c.1013A>G, p.His338Arg	ND	**12**	c.1559delT, p.Leu520Argfs*86	0	1
	11	c.1307A>G, p.Tyr436Cys	NR	**12**	c.1559delT, p.Leu520Argfs*86	0	1
	**12**	c.1559delT, p.Leu520Argfs*86	0	**12**	c.1559delT, p.Leu520Argfs*86	0	2
Childhood	5	c.407G>A, p.Arg136His	33.4	**12**	c.1559delT, p.Leu520Argfs*86	0	2
	6	c.572A>G, p.Glu191Gly	NR	**12**	c.1559delT, p.Leu520Argfs*86	0	1^a^
	9	c.979T>C, p.Phe327Leu	72	**12**	c.1559delT, p.Leu520Argfs*86	0	1
	**12**	c.1559delT, p.Leu520Argfs*86	0	*7*	*c*.*787T>C*, *p*.*Tyr246His*	SNP	1
Odonto	4	c.211C>T, p.Arg71Cys	0	*7*	*c*.*787T>C*, *p*.*Tyr246His*	SNP	2
	6	c.550C>T, p.Arg184Trp	0.6	WT		-	2
	6	c.550C>T, p.Arg184Trp	0.6	*7*	*c*.*787T>C*, *p*.*Tyr246His*	SNP	1
	9	c.979T>C, p.Phe327Leu	72	**12**	c.1559delT, p.Leu520Argfs*86	0	1
	10	c.1130C>T, p.Ala377Val	NR	WT		-	1
	*7* 10	*c*.*787T>C*, *p*.*Tyr246His* c.1144G>A, p.Val382Ile	SNP 0	*7*	*c*.*787T>C*, *p*.*Tyr246His*	SNP	1
	12	c.1375G>A, p.Val459Met	NR	WT		-	1
Total							35

^a^Case shifted from odonto to childhood type as the patient aged.

SNP of “c.787T>C, p.Tyr246His” displayed in italics. “c.1559delT, p.Leu520Argfs*86” is shown by bold letters.

ND, mutation type was not described; NR, mutation type was reported but no residual activity was detected.

Table 3 shows the inheritance patterns of the 35 probands. Inheritance is AR in 26 (74%) patients and AD in nine (26%). In the non-odonto type HPP, significantly more patients had AR inheritance (96%) than AD inheritance (4%) (*P*<0.0001). In the odonto-type HPP, AD mutations were detected in eight cases (89%), with significantly fewer patients demonstrating AR inheritance (11%) (*P*<0.05).

**Table 3:** Comparison of inheritance patterns between non-odonto and odonto types.

Phenotype	*n*	Autosomal dominant	Autosomal recessive	*p* value
Non-odonto	26	1 (4%)	25 (96%)	<0.0001
Odonto	9	8 (89%)	1 (11%)	0.020
**Total**	**35**	**9 (26%)**	**26 (74%)**	**0.004**

### Dental manifestations

The first dental visit for patients with severe-type HPP (perinatal severe and infantile types) was earlier than that for patients with the other phenotypes (Table 1). The average age at the first dental visit in patients with odonto-type HPP (3.5 ± 0.6 years) was significantly younger than that for patients with childhood-type HPP (6.7 ± 1.4 years; *P*<0.05).

**Fig 2:**
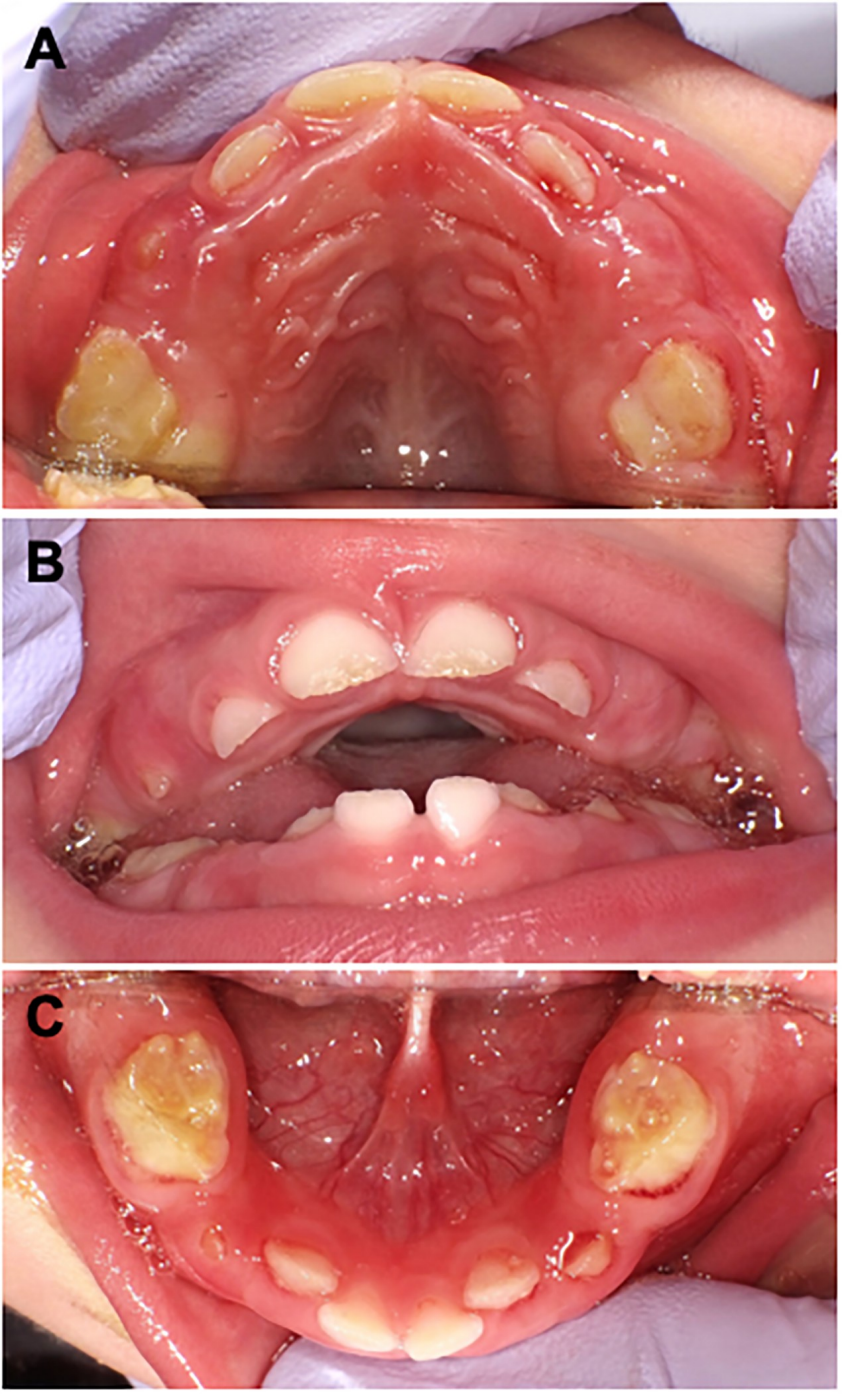
Intraoral photographs of an 18-month-old boy diagnosed with perinatal severe-type HPP. A. Maxillary occlusal view. B. Frontal view. C. Mandibular occlusal view. Typical findings of occlusal problems such as high-arched palate (A), open bite (B), crowding (C), and hypomineralisation of teeth (A–C) were present.

Spontaneous early exfoliation or extraction of primary teeth (caused by preservation difficulties) was reported in 36 (69%) cases, including all 16 (100%) patients with the odonto type; this finding was significant compared with the loss of dentition among the non-odonto types (*n* = 20/36 patients; 56%) (*P*<0.05) (Table 4). However, 42% of non-odonto-type cases (15/36 patients) showed tooth hypomineralisation, which was significantly higher than that found for the odonto type (0%; *P*<0.05). Fig 2 shows representative intraoral photographs of one of the non-odonto type cases (perinatal severe type with ERT), in which both tooth hypomineralisation and malocclusion were recognised. Occlusal problems (e.g., open bite or crowding) and skeletal problems of the maxilla and mandibular bones (e.g., thin jaw bone or high-arched palate) were reported predominantly in the non-odonto type.

**Table 4:** Dental problems associated with HPP.

Dental problems	Non-odonto (*n* = 36)	Odonto (*n* = 16)
	Primary dentition (*n* = 21) Mixed or permanent dentition (*n* = 15)	Primary dentition (*n* = 11) Mixed or permanent dentition (*n* = 5)
Experience of early exfoliation of primary teeth (<4 years)	20 (56%)	16^a^ (100%)
Periodontal condition		
-Experience of primary teeth mobility	20 (56%)	13 (81%)
-Permanent teeth mobility	4 (11%)	0 (0%)
-Loss of permanent teeth	2 (6%)	0 (0%)
Hypomineralisation	15^a^ (42%)	0 (0%)
-Enamel	15^a^ (42%)	0 (0%)
-Dentin	7 (19%)	0 (0%)
Eruption disturbance	8 (22%)	2 (13%)
Congenital missing tooth	3 (8%)	1 (6%)
Submerged tooth	3 (8%)	1 (6%)
Occlusion	13 (36%)	3 (19%)
-Open bite	5 (14%)	0 (0%)
-Crowding	4 (11%)	0 (0%)
-Anterior or posterior crossbite	2 (6%)	2 (13%)
-Others	2 (6%)	1 (6%)
Thin jaw bone	6 (17%)	1 (6%)
High arched palate	3 (6%)	0 (0%)

^a^The rate of the category is significantly higher than that of another category (*P*<0.05).

HPP, hypophosphatasia

Two cases in the non-odonto type (childhood and infantile types with ERT) experienced loss of permanent teeth, and four cases in the non-odonto type (perinatal benign infantile and childhood types without ERT, and childhood type with ERT) showed severe periodontitis and early exfoliation in primary dentition. In addition, early exfoliation of the primary teeth and treatment with ERT were not correlated (S1 Table).

### Correlation with genotype and dental phenotype

Most of the patients with odonto-type HPP (88.9%; Table 2) had heterozygous mutations, and most (96.2%) of the non-odonto-type patients had homozygous or compound heterozygous mutations. In addition, only one odonto-type HPP patient had a compound heterozygous mutation of “c.1559delT”; however, most of the non-odonto type (88.4%) patients had one or two “c.1559delT” mutations. Heterozygous mutations of “Arg167Trp” (c.550C>T, p.Arg184Trp in exon 6) were detected in three odonto-type cases, and the residual ALP activity levels in these patients were low (Table 2). The “Tyr263His” (c.787T>C, p.Tyr246His in exon 7) single-nucleotide polymorphism (SNP) was reported in four patients with odonto-type HPP and one with patient with childhood-type HPP. Furthermore, of these five SNP cases, four (three odonto-type and one childhood-type) had heterozygous mutations of c.787T>C, and one odonto-type case had a homozygous mutation of c.787T>C with an additional heterozygous mutation of c.1144G>A (p.Val382Ile in exon 10).

## Discussion

Patients diagnosed with severe-type HPP (perinatal severe or infantile types) generally die before the emergence of primary teeth in the oral cavity. Three years ago, the use of ERT was introduced in Japan, which drastically improved the prognosis of patients with HPP, including those with severe-type HPP [19–25], and increased the opportunities for these patients to receive dental management later, when necessary [26,27]. When we performed this same survey 5 years ago exploring the dental features of patients with HPP, there was little information regarding perinatal-severe type HPP^10^, even though this type of HPP accounted for 17% of the total number of patients with HPP in the present survey. Patients with perinatal-severe HPP in our survey were all referred by medical doctors for dental management. The average age at their first dental examination was 1.8 years; primary incisors, particularly mandibular primary incisors, which are frequently lost in HPP, have usually erupted by the age of 1.5 years [28]. Few reports regarding dental problems in patients with severe-type HPP have been published, and this survey revealed dental conditions not yet clarified for this cohort in the literature [26.27]. Because patients with severe-type HPP are usually diagnosed before birth, these patients should receive dental management and oral hygiene instruction before tooth eruption to maintain good periodontal conditions. The mandibular primary central incisors are usually the first primary teeth to emerge, roughly around 8 months of age [28]; therefore, a dental visit before this period should be recommended if medical conditions are to be sufficiently improved to avoid early exfoliation of the primary teeth.

Dental symptoms are often the first clue for an early diagnosis for some patients with HPP^3^. Early tooth exfoliation can sometimes lead to a diagnosis of mild-type HPP, such as odonto and childhood types [11,12]. In this study, the mean age at diagnosis for patients with odonto-type HPP was significantly younger than that for patients diagnosed with childhood-type HPP. Two patients shifted from the odonto type to the childhood type because of the emergence of systemic bone symptoms as they aged. Indeed, patients with odonto-type HPP are at risk for progression to childhood- or adult-type HPP with age, and thus an early diagnosis and medical management are important for these patients [11,18]. In the present study, the mean serum ALP values for odonto-type cases were significantly higher than those for perinatal-severe-, perinatal-benign-, and infantile-type cases. Considering the early detection of patients with odonto-type HPP, we need to monitor ALP serum levels in children with suspected HPP, which is higher than that of adults [13]. Teeth might be more sensitive to serum ALP levels than other calcified tissues in patients with odonto-type HPP. In mild-type patients with one or two exfoliated teeth before medical diagnosis of HPP, a relevant differential diagnosis of dental trauma versus HPP is difficult [12,27]. In our study, 80% of patients reported mobility of the primary teeth, which may be exfoliated with light pressure. Dentists need to check the mobility of other residual primary incisors when they encounter early exfoliation of primary teeth with long roots, which is a typical manifestation of HPP.

In the present study, one patient with a compound heterozygous mutation shifted from the odonto-type to childhood-type group at 7 years and 8 months. Given that most of the cases in the odonto-type group had heterozygous mutations, it is reasonable to speculate that patients with heterozygous mutations have odonto-type HPP, whereas those with compound heterozygous mutations are likely to shift toward childhood-type HPP as they grow. Indeed, there is presently one odonto-type patient (3 years and 5 months) with a compound heterozygous mutation, who we predict may shift to childhood-type HPP as they grow; it will be important to carefully monitor this child. Furthermore, there are some cases who have been diagnosed as childhood-type who could have been classified as odonto-type if they had been diagnosed based on the findings of early exfoliation of primary teeth in the absence of systemic problems. Taken together, a genetic analysis is important not only for a definitive diagnosis of the condition but also perhaps for a prediction of prognosis.

Over 360 mutations in the *ALPL* gene have been registered in “The Tissue Nonspecific Alkaline Phosphatase Gene Mutations Database” (http://www.sesep.uvsq.fr/03_hypo_mutations.ph). The “T1559del” (c.1559delT, p.Leu520Argfs*86 in exon 12) and “Phe310Leu” (c.979T>C, p.Phe327Leu in exon 9) mutations are more commonly found in Japanese patients with HPP [13,15–17], which is consistent with our present results. In particular, half of the perinatal severe-type HPP cases had “c1559delT” homozygous mutations. Previous studies have reported that the enzyme activity is almost completely lost in patients with the “c.1559delT” mutant TNSALP protein, and that this mutation is associated with lethal types of HPP [13,15–17]. In this study, patients with this homozygous mutation had perinatal- or infantile-type HPP. We reinvestigated all of the cases with childhood-type HPP for whom this mutation pattern was identified, and found that one patient with a heterozygous “c.1559delT” mutation shifted from odonto-type to childhood-type with age. In addition, one odonto-type HPP patient had a “c.1559delT” compound heterozygous mutation. The enzyme activity of “c.1559delT” is extremely low, and we must pay attention to ALP levels in mild-type HPP patients bearing this compound heterozygous “c.1559delT” mutation. We must also consider the progression of localised dental symptoms to whole-body changes, which will alter a diagnosis from odonto-type to childhood- or adult-type. We consider the “c.1559delT” mutation to be a marker for the potential progression of HPP in patients with odonto-type HPP. The frequency of heterozygous “c.1559delT” mutations among healthy Japanese is estimated to be about 1 in 480 [17]. Yet, the number of odonto-type HPP cases in this study was fewer than that of severe-type HPP. There might be many mild cases (odonto, childhood and adult type HPP) among the population that are, as yet, undiagnosed. Dental symptoms such as “early exfoliation of primary teeth before 4 years old” offer an important opportunity for dentists to diagnose HPP.

The “Phe310Leu” (c.979T>C, p.Phe327Leu in exon 9) mutation was detected in five cases (14%) spanning the perinatal-benign, infantile, childhood and odonto types. All of these mutations were compound heterozygous mutations of “Phe310Leu” and “c.1559delT”. “Phe310Leu” is the second-most frequent mutation among Japanese patients, and the “Phe310Leu”/“c.1559delT” combination is common [13,17]. Patients with the “Phe310Leu” mutation have some residual enzyme activity, and a relatively mild type of HPP [13,17]. In HPP patients with a heterozygous mutation of “c.1559delT”, who have an almost complete loss of enzyme activity, the “Phe310Leu” mutation contributes to a relatively mild HPP phenotype [13,17]. Indeed, in our study, none of the perinatal-severe-type HPP patients had a “Phe310Leu” mutation.

There were more patients with AD inheritance than AR inheritance in the odonto-type group. Patients with mild forms of HPP, such as odonto-type, generally have AD inheritance, which is consistent with those surveyed in the USA [3]. Heterozygous mutations “c.211C>T, p.Arg71Cys in exon 4” and “550C>T, p.Arg184Trp in exon 6” have been reported in odonto-type cases [3]. However, specific compound heterozygous or homozygous mutations are associated with specific phenotypes: “c.211C>T, p.Arg71Cys in exon 4” with infantile; “550C>T, p.Arg184Trp in exon 6” with perinatal; “c.1130C>T, p.Ala377Val in exon 10” with infantile; “c.1144G>A, p.Val382Ile in exon 10” with perinatal; and “c.1375G>A, p.Val459Met in exon 12” with childhood and infantile HPP [30–34]. The “c.211C>T, p.Arg71Cys in exon 4”, c.550C>T, p.Arg184Trp in exon 6 and “c.1144G>A, p.Val382Ile in exon 10” mutations, detected in six cases, were all heterozygous mutations in odonto-type HPP. The residual ALP activity was almost lost in these cases [35–37], with a dominant-negative effect reported in 30% to 36.7% of cases [14,36,38]. This dominant-negative effect first causes dental symptoms in children, yet parents of children with odonto-type HPP did not report dental manifestations; these mutations possibly have low penetrance [1].

The “Tyr263His” (c.787T>C, p.Tyr246His in exon 7) SNP was reported in four odonto-type patients and one childhood-type patient (36% of HPP patients in our clinic) who had heterozygous mutations of *ALPL*. This SNP was shown to decrease the catalytic properties of TNSALP and reduce the bone mineral density in older Japanese women [39,40]. This SNP has also been reported in a Chinese family with odonto-type HPP; the study suggested that this SNP might interfere with ALP activity and could be responsible for odonto-type HPP [41]. Moreover, the compound heterozygous mutations of “Tyr263His” and “Ser445Pro” are associated with perinatal-severe-type HPP [31]. However, another study reported that this SNP is a casual mutation in linkage disequilibrium with haplotype E, which together act as an aggravating factor that has resulted in haploinsufficiency in a family with European ancestry [14]. Taken together, these findings suggest that this SNP influences the occurrence of mutations of TNSALP on other alleles and that compound heterozygosity between the pathogenic mutation on one allele and this SNP on the other leads to a mild type of HPP [1].

Early exfoliation of primary teeth has been the main dental manifestation of HPP. In this survey, however, we noted cases of severe periodontitis in permanent dentition, as well as other oral problems among patients with HPP. Indeed, as the severity of an HPP patient’s systemic condition increases, the loss of mineralisation in the teeth and jaw worsens. We did not find an association between treatment with ERT and early exfoliation of the primary teeth. Patients with perinatal-severe-type HPP generally begin ERT just after birth, whereas patients with childhood- or adult-type HPP do not receive ERT after the eruption of permanent teeth. Improvements in skeletal hypomineralisation in the perinatal-benign form of HPP can occur in utero during pregnancy due to maternal circulating ALP levels, and such tooth mineralisation before birth might be reflected by these placental ALP levels [42]. However, most permanent teeth are mineralised soon after birth. The timing of ERT may also influence tooth mineralisation. For many with occlusal problems, such as an open bite or a high-arched palate, who are considered to have severe-type HPP, these changes can lead to difficulty in swallowing and mastication. Total dental management, including the persistence of occlusal problems, is important especially for perinatal and infantile types, depending on the severity of HPP.

The present study revealed distinct differences in the oral manifestations of HPP between odonto-type and non-odonto-type HPP. In odonto-type cases, early exfoliation of the primary teeth due to disrupted cementum formation was common. On the contrary, in the non-odonto-type cases, tooth hypomineralisation, such as weakening of the enamel, dentine, cementum and jaw bone, was associated with low serum ALP levels. Hypomineralisation of the jaw bone can cause many occlusal problems. In the present study, we could not establish criteria to evaluate occlusal problems. Patients with severe-type HPP often present with dysphasia or respiratory difficulties, which can result in occlusal problems, such as a high-arched palate. It will be important to establish diagnostic criteria for occlusion among HPP patients in future studies. Furthermore, it is worth acknowledging that dental problems that occur in the primary dentition could cause similar issues in the permanent dentition. Dental management of enamel and dentine hypomineralisation and malocclusion, in addition to periodontal conditions, are necessary for patients with non-odonto-type HPP. Another limitation of this study was that our findings were based on questionnaire surveys within the dental field, which did not always include direct medical information, such as radiographic examinations or genetic analyses.

Previous studies have reported that odonto-type HPP cases sometimes shift to childhood- or adult-type HPP over time [7,12]. Therefore, the identification of odonto-type cases is important for early diagnosis and continuous medical management. Genetic testing is also necessary to confirm a diagnosis of HPP, particularly for patients with odonto-type HPP, as this is the mildest type. Genetic patterns could be used to predict the prognosis of odonto-type HPP and aid in prevention and treatment planning. Indeed, those with an AR inheritance pattern or those with a single “c.1559delT” mutation might later progress to a more severe type with a bone condition.

In summary, the genetic and dental manifestations of odonto-type and non-odonto-type HPP cases are different. Medical and dental practitioners should collaborate to provide patients with early diagnoses and to design an appropriate approach for the treatment of associated dental problems, such as hypomineralisation of the tooth and jaw bone.

## Supporting information

S1 Table Enzyme replacement therapy in patients with early exfoliation of primary teeth. (TIF)

S1 Fig Contents of the first questionnaire. (TIF)

S2 Fig Contents of the second questionnaire. (TIF)

S1 Data (XLSX)
