# Supplementary figures and images for: Japanese nationwide survey of hypophosphatasia reveals prominent differences in genetic and dental findings between odonto and non-odonto types
Source: PLoS One. 2019 Oct 10;14(10):e0222931. doi: 10.1371/journal.pone.0222931 (PMC6786601; doi:10.1371/journal.pone.0222931)

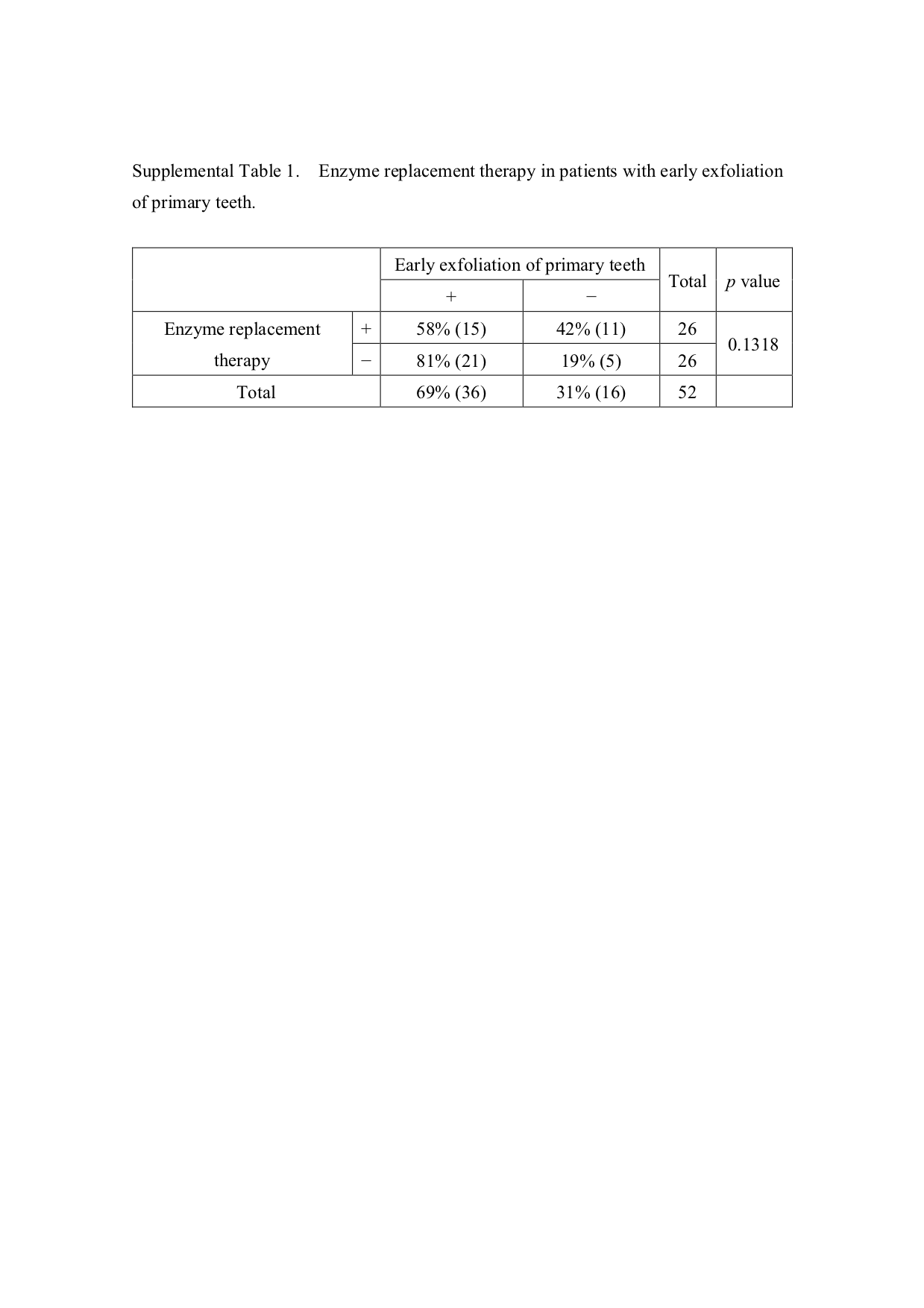

Supplement: S1 Table — (TIF) [file pone.0222931.s001.tif]

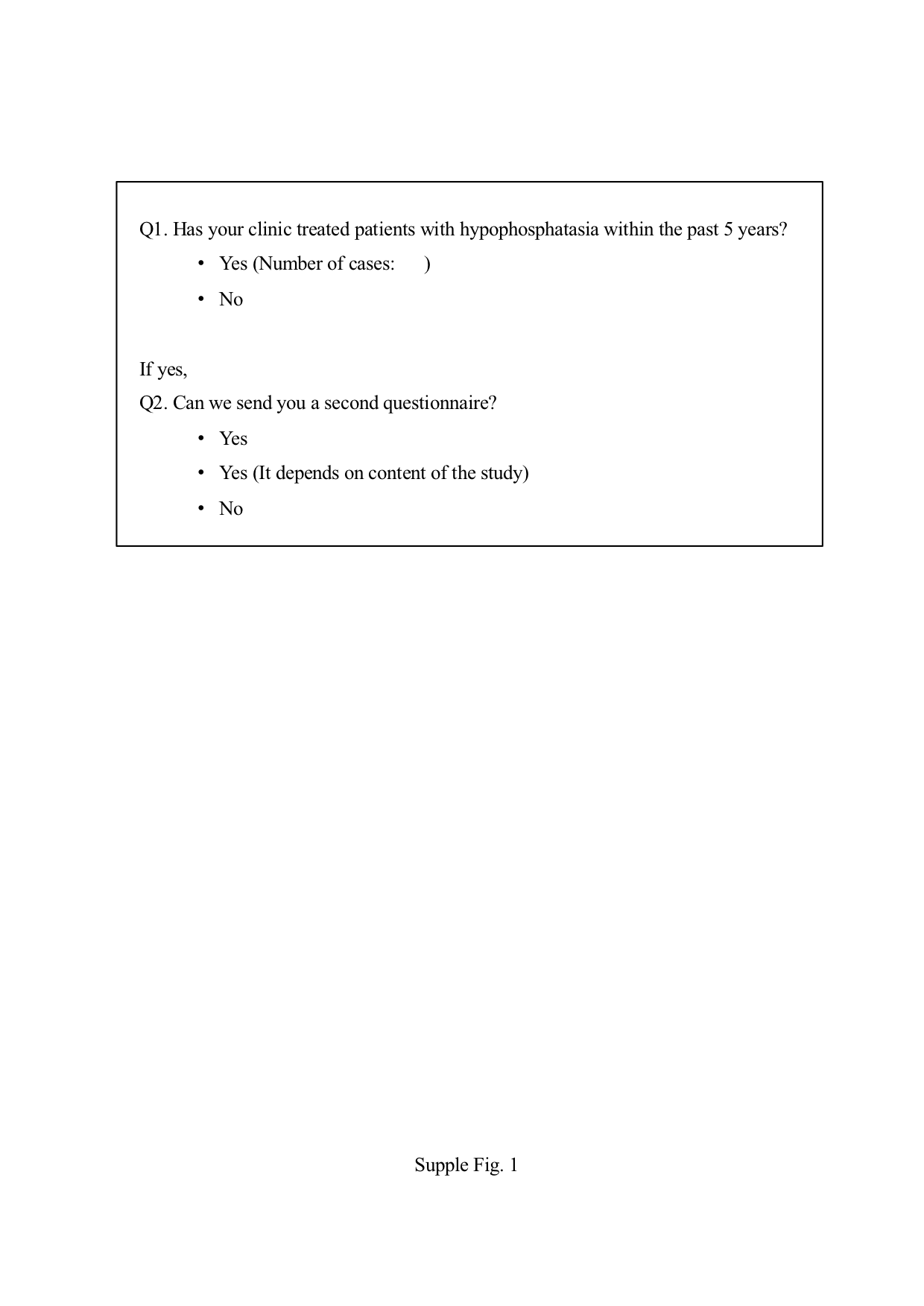

Supplement: S1 Fig — (TIF) [file pone.0222931.s002.tif]

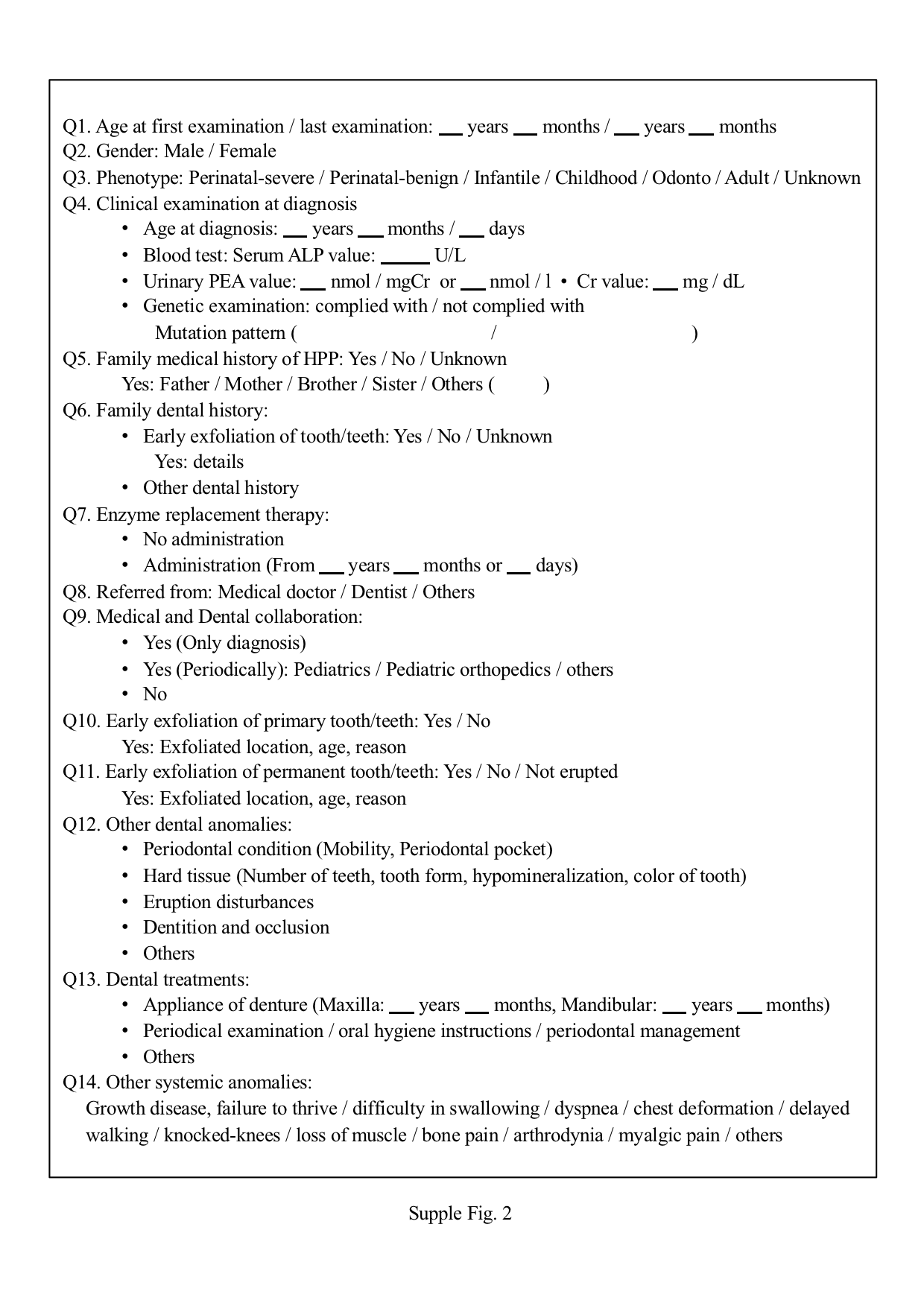

Supplement: S2 Fig — (TIF) [file pone.0222931.s003.tif]
